# Supplementary material for: Opuntia dillenii Haw. Polysaccharide Promotes Cholesterol Efflux in THP-1-Derived Foam Cells via the PPARγ-LXRα Signaling Pathway
Source: Molecules. 2022 Dec 7;27(24):8639. doi: 10.3390/molecules27248639 (PMC9781717; doi:10.3390/molecules27248639)
Supplement: Supplementary file 1 [file molecules-27-08639-s001.zip › molecules-2023506-Supplementary.pdf]

Supplementary **Table** S1 Primer sequences

Gene		Primer sequences
GAPDH	Forward	5′–CTGACTTCAACAGCGACACCCA–3′
	Reverse	5′–CCACCCTGTTGCTGTAGCCA–3′
	Probe	5′–CCAGCCCCAGCGTCAAAGGT–3′
ABCA1	Forward	5′–CTCCTCCACCCAAATCTACCA–3′
	Reverse	5′–GCATCTTCCTCAGTGCCAT–3′
	Probe	5′–ATGCCCGCAGACAATACGAGACA–3′
ABCG1	Forward	5′–AAAAGTCTGCAATCTTGTGCCAT–3′
	Reverse	5′–TCCCGAACCGCTCTCACC–3′
	Probe	5′–ATCACCGTACTCGCCGGATGCAA–3′
SR–BI	Forward	5′–GCGACTACATCGTCATGCCCAAC–3′
	Reverse	5′–GATCTCACCCACAGTGCGGTTC–3′
	Probe	5′–CACCGCCGCACCCAAGACCA–3′
PPARγ	Forward	5'–AGATCTCCAGTGATATCGACCA–3'
	Reverse	5'–TGTCTTTCCTGTCAAGATCGC–3'
	Probe	5'–ATCCAGAGTCCGCTGACCT–3'
PPARα	Forward	5'–AAACAAATGCCAGTATTGTCGATT–3'
	Reverse	5'–CTTTCTCAGATCTTGGCATTCGTC–3'
	Probe	5'–ACATCCCGACAGAAAGGCACT–3'
LXRα	Forward	5'–GCTCCTTTTCTGACCGGCTT–3'
	Reverse	5'–TTGGCAAAGTCTTCCCGGTT–3'
	Probe	5'–CACTCCCAGGGTTGTACCTCCG–3'
